# Supplementary material for: Dynamic transcriptomic profiles of zebrafish gills in response to zinc supplementation
Source: BMC Genomics. 2010 Oct 11;11:553. doi: 10.1186/1471-2164-11-553 (PMC3091702; doi:10.1186/1471-2164-11-553)
Supplement: Additional file 2 — Interactive Direct Interaction Network representing the molecular interactions between zinc, copper, iron, calcium and proteins encoded by transcripts changed by zinc supplementation. Mini web-site containing index.html and hyperlinked pages in subdirectory describing a Direct Interaction Network automatically generated based on curated interactions contained within the proprietary PathwayArchitect database. Ovals represent proteins and the circles symbolize metal ions. Objects are coloured by their abundance in zebrafish at the time-point they were significantly different from the control is a scale from -4 fold (dark green) to +4 fold (dark red). Where significant differences were found at more than one time-point, the colour overlay shows expression at the first instance. Dark blue squares denote 'binding', and light blue squares 'expression'; green squares stand for 'regulation', green diamonds for 'metabolism', and green circles for 'promoter binding'. Arrow heads indicate directionality of the interaction where annotated. All nodes and edges can be further interrogated by selecting the relative area of the image. [file 1471-2164-11-553-S2.zip › PathwayArchitect Zn xs DIN/534152.html]

# TRANSPORT:

|  |  |
| --- | --- |
| Type | TRANSPORT |
| Effect | None |


---

|  |  |
| --- | --- |
| Score | 0 |


---

|  |  |
| --- | --- |
| Reference Count | 5 |


---

|  |  |
| --- | --- |
| Mechanism | Unknown |


---

|  |  |
| --- | --- |
| Reference:0 || Sentence | "After pre-incubation in Ca2+-free medium, re-introduction of Ca2+ (1.3 mmol/l) elicited beta-End-IR release." |
| PMID | 3018594 |
| Year | 1986 |
| Species | Rat |
| Journal | Naunyn Schmiedebergs Arch Pharmacol |
| RefScore | 0 |
| Source | PArchNLP |
  |
|  |  |


---

|  |  |
| --- | --- |
 Reference:1 || Sentence | "These results suggest a specific stimulatory effect of AVP and osmolarity and a negative influence of extracellular Ca2+ on atrial spontaneous Ir-ANP release." |
| PMID | 1667640 |
| Year | 1991 |
| Species | Rat |
| Journal | Eur J Pharmacol |
| RefScore | 0 |
| Source | PArchNLP |
  ||  |  |


---

|  |  |
| --- | --- |
 Reference:2 || Sentence | "The K(+)-evoked release of ir-alpha-NEO was Ca2(+)-dependent, and the veratridine stimulation was abolished by 1 microM tetrodotoxin." |
| PMID | 1980349 |
| Year | 1990 |
| Species | Rat |
| Journal | Neuropeptides |
| RefScore | 0 |
| Source | PArchNLP |
  ||  |  |


---

|  |  |
| --- | --- |
 Reference:3 || Sentence | "The presence of Ca2+ in the incubation medium was required for stimulation of the release of the immunoreactive beta-endorphin-like peptide (IR-beta-EP) from the dispersed cells of the neurointermediate lobe of rat pituitary gland by adenosine 3',5'-monophosphate (cAMP) analogs, a phosphodiesterase inhibitor, L-isoproterenol, cholera toxin and forskolin." |
| PMID | 2582172 |
| Year | 1985 |
| Species | Rat |
| Journal | Jpn J Pharmacol |
| RefScore | 0 |
| Source | PArchNLP |
  ||  |  |


---

|  |  |
| --- | --- |
 Reference:4 || Sentence | "The synaptic origin of CRF-IR release in the amygdala was determined in vitro by assessing the Ca2+ dependency of 4-aminopyridine stimulated CRF-IR release from tissue preparations of rat amygdala." |
| PMID | 7643193 |
| Year | 1995 |
| Species | Rat |
| Journal | J Neurosci |
| RefScore | 1 |
| Source | PArchNLP |
  |


---

|  |  |
| --- | --- |
